# Proteomics analysis of deep fascia in acute compartment syndrome

**DOI:** 10.1371/journal.pone.0305275

**Published:** 2024-07-01

**Authors:** Haofei Wang, Yan Liu, Sujuan Xu, Tao Wang, Xiaojun Chen, Huiyang Jia, Qi Dong, Heng Zhang, Shuai Wang, Huijie Ma, Zhiyong Hou

**Affiliations:** 1 Department of Orthopaedic Surgery, Third Hospital of Hebei Medical University, Shijiazhuang, Hebei Province, China; 2 Key Laboratory of Biomechanics of Hebei Province, Shijiazhuang, Hebei Province, China; 3 Orthopaedic Research Institution of Hebei Province, Shijiazhuang, Hebei Province, China; 4 Department of Endocrinology, Third Hospital of Hebei Medical University, Shijiazhuang, Hebei Province, China; 5 Department of Nephrology, Third Hospital of Hebei Medical University, Shijiazhuang, Hebei Province, China; 6 Department of Physiology, Hebei Medical University, Shijiazhuang, Hebei Province, China; 7 NHC Key Laboratory of Intelligent Orthopaedic Equipment, Third Hospital of Hebei Medical University, Shijiazhuang, Hebei Province, China; Ningbo University, CHINA

## Abstract

Acute compartment syndrome (ACS) is a syndrome in which local circulation is affected due to increased pressure within the compartment. We previously found in patients with calf fractures, the pressure of fascial compartment could be sharply reduced upon the appearance of tension blisters. Deep fascia, as the important structure for compartment, might play key role in this process. Therefore, the aim of the present study was to examine the differences in gene profile in deep fascia tissue in fracture patients of the calf with or without tension blisters, and to explore the role of fascia in pressure improvement in ACS. Patients with lower leg fracture were enrolled and divided into control group (CON group, n = 10) without tension blister, and tension blister group (TB group, n = 10). Deep fascia tissues were collected and LC-MS/MS label-free quantitative proteomics were performed. Genes involved in fascia structure and fibroblast function were further validated by Western blot. The differentially expressed proteins were found to be mainly enriched in pathways related to protein synthesis and processing, stress fiber assembly, cell-substrate adhesion, leukocyte mediated cytotoxicity, and cellular response to stress. Compared with the CON group, the expression of Peroxidasin homolog (PXDN), which promotes the function of fibroblasts, and Leukocyte differentiation antigen 74 (CD74), which enhances the proliferation of fibroblasts, were significantly upregulated (*p* all <0.05), while the expression of Matrix metalloproteinase-9 (MMP9), which is involved in collagen hydrolysis, and Neutrophil elastase (ELANE), which is involved in elastin hydrolysis, were significantly reduced in the TB group (*p* all <0.05), indicating fascia tissue underwent microenvironment reconstruction during ACS. In summary, the ACS accompanied by blisters is associated with the enhanced function and proliferation of fibroblasts and reduced hydrolysis of collagen and elastin. The adaptive alterations in the stiffness and elasticity of the deep fascia might be crucial for pressure release of ACS.

## Introduction

Acute compartment syndrome (ACS) is a syndrome in which local circulation is affected due to increased pressure within the compartment. The pathophysiological alteration is the accumulation of blood or tissue fluid in the compartment, leading to progressive increase of contents and pressure in compartment, eventually result in ischemic necrosis of muscles and nerves [1]. Delayed treatment may lead to severe consequences such as limb dysfunction.

The etiology, diagnosis and treatment of ACS are still challenging since controversies still exist, regarding the pressure threshold for the diagnosis of ACS, the optimal time for fascial compartment dissection, the location of intra-compartment pressure measurement, and the method of measurement [2]. It has been widely believed that early fasciotomy and reduction surgery could prevent the development of ACS [3]. Prophylactic fasciotomy reduction has been also advocated. However, effects are not satisfactory by causing overtreatment, prolonged hospitalization and increasing economic burden on individual and the society [4].

Hou et al [5] has found that, patients with lower leg fractures had a sharp reduction in fascial compartment pressure, pain, and numbness after the development of blisters on the skin of the affected limb, suggesting that the limb with a predominantly skeletal injury may release the intrinsic pressure when the fascial compartment pressure reaches a certain level, thus avoiding the serious consequences of ACS. From this clinical phenomenon, Hou et al therefore developed the hypothesis of "myofascial self-release law" [5].

The fascial compartment is formed mainly by muscles, blood vessels and nerve fibers, and all these tissues are wrapped and contained by deep fascia. As an important structure, it is highly likely that deep fascia plays an essential role in ACS. However, there is very little data available about the contributions of deep fascia to the fate of ACS. Therefore, in the present study, we aimed to check the altered gene profile in deep fascia tissue from patients with fracture of the lower leg with blisters on the skin, and try to explore the role of fascia plays in pressure release during ACS, which may provide new evidences for ACS diagnosis, treatment decision, and prognosis evaluation.

## Materials and methods

### Experimental design and statistical rationale

Patients with fracture of the lower leg were enrolled from the Third Hospital of Hebei Medical University from May 2021 to November 2021. The included patients were assigned to two groups, fracture with tension blister group (TB group, n = 10) and fracture without blister group (control group, CON group, n = 10) according to below criteria.

The inclusion criteria for the TB group were as follows: (1) patients with closed fractures of the calf who presented with the clinical manifestations of typical ACS in the absence of external restraints such as casts or splints, including abnormal swelling and paresthesia in the affected limb, severe pain that was inconsistent with the original injury, passive traction pain, which relieved progressively with the appearance of tension blisters, (2) adult patients (≥ 18 years old), and (3) no comorbidity was present at the time of admission. And the inclusion criteria for the CON group were the following: 1) patients with closed fractures of the calf and no tension blisters on the skin. (2) Adult patients (≥18 years of age), and (3) no comorbidities at the time of admission.

Exclusion criteria: (1) pathological fractures, (2) artery injuries which affects the blood supply of the affected limb, (3) crush syndrome, (4) with other diseases such as diabetes, infectious diseases, autoimmune diseases, malignancy, heart failure, and renal and hepatic functional impairment.

The deep fascia tissues of the lower legs were collected during internal fixation of fracture and immediately stored at -80°C until the proteins were extracted. The statistical methods for analysis were described in the following sections.

### Ethics

The human studies reported in our manuscript abide by the Declaration of Helsinki principles. The study was approved by the Medical Ethics Committee of the Third Hospital of Hebei Medical University (approval number:S2020-024-1) and the informed consent forms were signed by all patients.

### Protein extraction

The sample was grinded with liquid nitrogen into cell powder and then transferred to a 5ml centrifuge tube. After that, four volumes of lysis buffer (1% SDS, 1% protease inhibitor cocktail) was added to the cell powder, followed by sonication three times on ice using a high intensity ultrasonic processor (Scientz, Ningbo, China). The remaining debris was removed by centrifugation at 12,000 g at 4°C for 10 min. Finally, the supernatant was collected and the protein concentration was determined with BCA kit (Thermo Fisher, 23225) according to the manufacturer’s instructions.

### Trypsin digestion

The protein sample was added with 1 volume of pre-cooled acetone, vortexed to mix, then added with 4 volumes of pre-cooled acetone and precipitated at -20°C for 2 h. The protein sample was then redissolved in 200 mM TEAB and ultrasonically dispersed. Trypsin was added at 1:50 trypsin-to-protein mass ratio for the first digestion overnight. The sample was reduced with 5 mM dithiothreitol for 60 min at 37°C and alkylated with 11 mM iodoacetamide for 45 min at room temperature in dark. Finally, the peptides were desalted by Strata X SPE column.

### LC–MS/MS analysis

The tryptic peptides were dissolved in solvent A (0.1% formic acid, 2% acetonitrile/in water), directly loaded onto a home-made reversed-phase analytical column with integrated spray tip (25-cm length, 100 μm i.d.) packed with 1.9 μm/120 Å ReproSil-PurC18 resins (Dr. Maisch GmbH, Ammerbuch, Germany). Peptides were separated with a gradient from 6% to 24% solvent B (0.1% formic acid in acetonitrile) over 70 min, 24% to 32% in 14 min and climbing to 80% in 3 min then holding at 80% for the last 3 min, all at a constant flow rate of 450 nL/min on a nanoElute UHPLC system (Bruker Daltonics, USA).

The peptides were subjected to capillary source followed by the timsTOF Pro (Bruker Daltonics, USA) mass spectrometry. The electrospray voltage applied was 1.75 kV. Precursors and fragments were analyzed at the TOF detector, with a MS/MS scan range from 100 to 1700 m/z. The timsTOF Pro was operated in parallel accumulation serial fragmentation (PASEF) mode. Precursors with charge states 0 to 5 were selected for fragmentation, and 10 PASEF-MS/MS scans were acquired per cycle. The dynamic exclusion was set to 30 s.

### Database search

The resulting MS/MS data were processed using MaxQuant search engine (v.1.6.15.0). Tandem mass spectra were searched against the human SwissProt database (20387 entries) concatenated with reverse decoy database. Trypsin/P was specified as cleavage enzyme allowing up to 2 missing cleavages. The mass tolerance for precursor ions was set as 20 ppm in first search and 5 ppm in main search, and the mass tolerance for fragment ions was set as 0.02 Da. Carbamidomethyl on Cys was specified as fixed modification, and acetylation on protein N-terminal and oxidation on Met were specified as variable modifications. FDR was adjusted to < 1%.

### Bioinformatics methods

Gene Ontology (GO), COG/EuKaryotic Orthologous Groups (KOG) functional classification statistics and Kyoto Encyclopedia of Genes and Genomes (KEGG) pathway analysis were performed on the differential proteins.

The GO analysis is a commonly used and productive method for annotating genes and gene products and for identifying the biological characteristics of high-throughput genome or transcriptome data [6]. GO annotation proteome was derived from the UniProt-GOA database (http://www.ebi.ac.uk/GOA/). Firstly, convert identified protein ID to UniProt ID and then map to GO IDs by protein ID. If some identified proteins were not annotated by UniProt-GOA database, the InterProScan [7] would be used to annotated proteins’ GO function based on protein sequence alignment method. Then proteins were classified by GO annotation based on three categories: biological process, cellular component and molecular function. For each category, a two-tailed Fisher’s exact test was employed to test the enrichment of the differentially expressed proteins against all identified proteins. Corrected p-value < 0.05 is considered significant.

The COG/KOG (https://www.ncbi.nlm.nih.gov/COG/) database is based on the phylogenetic relationship of encoded proteins of the complete genomes of bacteria, algae, and eukaryotes. The alignment could annotate a specific protein sequence to a specific COG, and each cluster of the COG is composed of orthologous sequences, which enables the identification of sequence functions [8]. Functional classification statistics of differentially expressed proteins were performed by COG/KOG database.

The KEGG is an open and collective database integrating genomes, biological pathways, diseases, drugs, and chemical substances [9]. KEGG database was used to identify enriched pathways. Firstly, using KEGG online service tool KAAS to annotated proteins’ KEGG database description, then map the annotation results on the KEGG pathway database using KEGG online service tool KEGG mapper. A two-tailed Fisher’s exact test was employed to perform the pathway enrichment significance analysis of the differentially expressed proteins against all identified proteins. The pathway with a corrected p-value < 0.05 was considered significant.

In addition, WoLF PSORT software was used for subcellular structure annotation of the proteins [10]. Wolfpsort is an updated version of PSORT/PSORT II for the prediction of eukaryotic sequences, which predicts the subcellular location on the basis of amino acid residues of the protein.

All differentially expressed proteins accession numbers or sequences were examined thoroughly by the STRING database (version 11.5) for convenient presentation and (Protein-protein Interaction) PPI analysis. Fetching all interactions that had a confidence score >0.7 (high confidence).The PPI network was constructed and visualized using Cytoscape (v3.9.1) [11]. The molecular complex detection (MCODE) plug-in in Cytoscape was used to screen the modules of the PPI network [12]. The inferred modules used the default settings with the degree cutoff = 2, node score cutoff = 0.2, K-core = 2, and max depth = 100. The GO analysis of the genes in each module was performed using the Database for Annotation Visualization and Integrated Discovery (DAVID), an online bioinformatics tool, to interpret the GO functions of those DEGs [13]. The visualization was plotted by https://www.bioinformatics.com.cn (last accessed on 10 Nov 2023), an online platform for data analysis and visualization [14].

ClueGO, a plug-in of Cytoscape, could classify non-redundant GO terms and visualize the functionally related genes in a clustered network [15]. We performed a GO analysis by utilizing the ClueGO (v2.5.9). The p-value <0.05 was identified as the significant GO term.

### Western blot

Proteins were extracted from deep fascia tissues. The equal amounts of proteins were isolated using SDS-PAGE and the protein was transferred to the PVDF membrane. The membranes were then incubated at room temperature for 24 h with following antibodies: Peroxidasin homolog (PXDN, 1:500, Abcam, Cambridge, UK), Leukocyte differentiation antigen 74 (CD74, 1:1000, Bioss, Beijing, China), Matrix metalloproteinase-9 (MMP9,1:1000, Abcam, Cambridge, UK), Neutrophil elastase (ELANE,1:500, Abcam, Cambridge, UK) and β-actin (1:10000, ProteinTech Group, Chicago, IL, USA). And β-actin was used as internal control. The optical density of bands was analyzed using ImageJ software.

### Statistical analysis

Python (version 3.7.6) was used to perform the statistical analyses. Student t-test was used to check the differences between groups, and *p* <0.05 was considered to be statistically different.

## Results

### Patients enrollment

**Fig 1:**
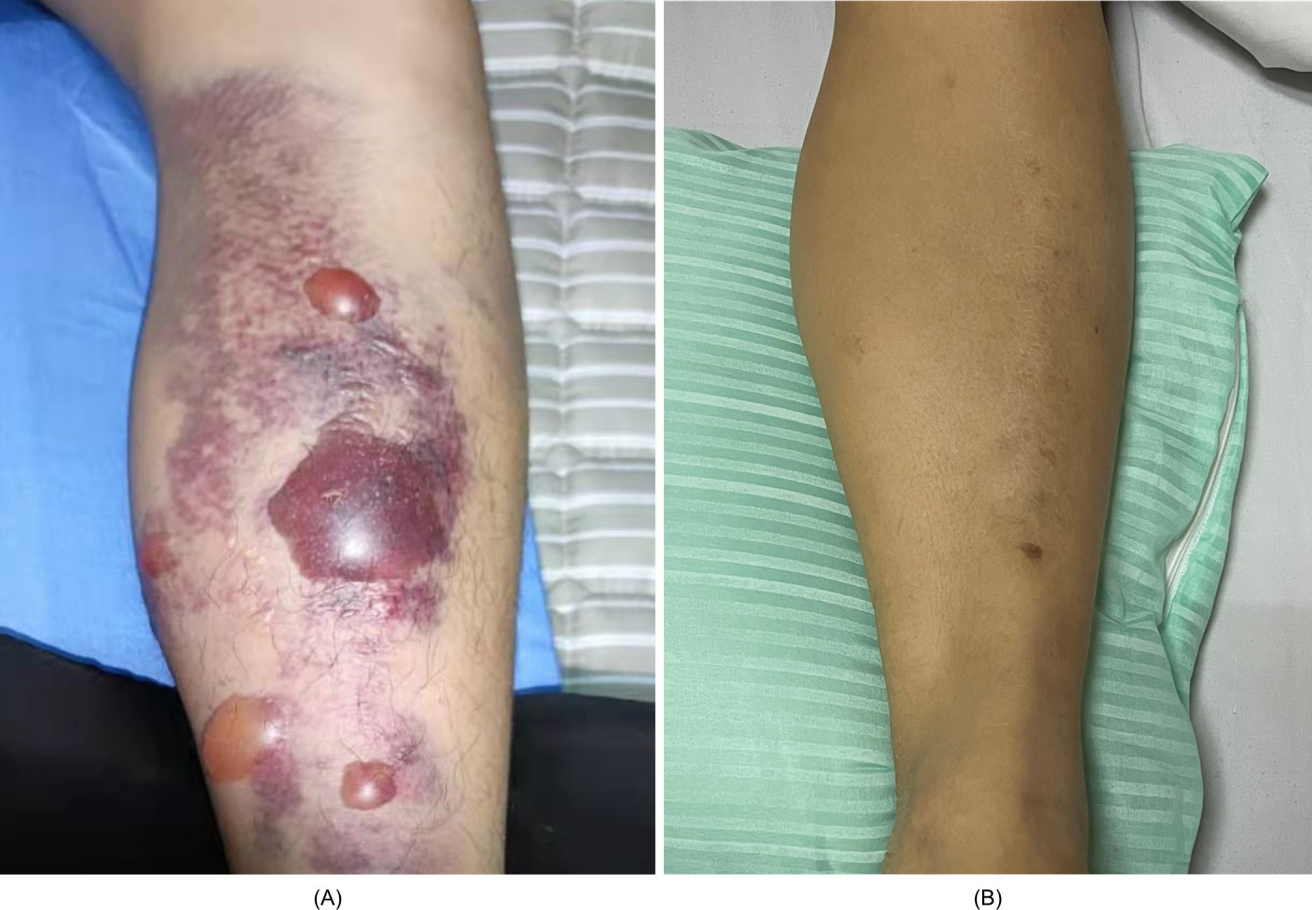
Images of patients with calf fractures. (A) The calf fracture patient with the appearance of blisters. (B) The calf fracture patient without blisters.

During the study period from May 2021 to November 2021, 10 patients with tension blister (Fig 1A) were included into TB group, and 10 patients without tension blister (Fig 1B) were included into CON group. The demographics and clinical data of the patients are presented in Table 1. There were no statistical differences in gender, age, cause of injury, or time from diagnosing to operation between these groups.

**Table 1:** Demographic and clinical features of the study cohort.

	Gender	Age (year, Mean ± SD)	Cause of Injury	Time from diagnosing to operation (hour, Mean ± SD)
CON	Female(5/10) Male(5/10)	51.50±13.02	Fall (6/10) Traffic accident (3/10) Collision (1/10)	105.60±9.87
TB	Female(3/10) Male(7/10)	48.60±12.33	Fall (5/10) Traffic accident (5/10)	108.10±14.13

TB, tension blister group; CON, control group; SD, standard deviation.

### Fascia proteomic profiles exhibited marked differences in two group of patients

**Fig 2:**
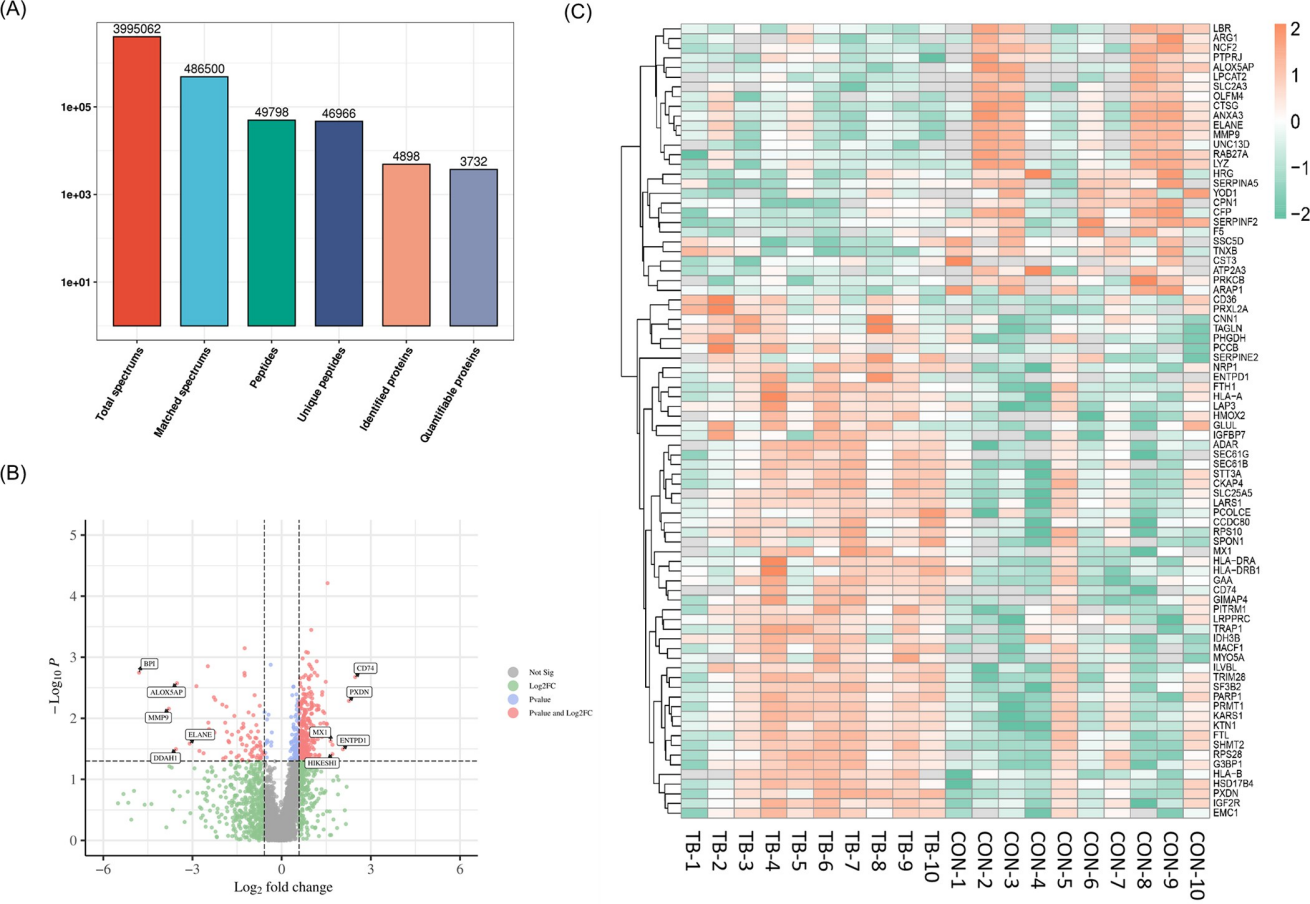
Protein identification results. (A) The total number of peptides and proteins identified. (B) Volcano plot for the comparison between the patients with and without blisters. The DEPs (fold change ≥ 1.5, P < 0.05) highlight in a pink color. The gene names of the five most significantly up-regulated DEPs and the five most significant down-regulated DEPs are marked on the plot. (C) Heatmap of the DEPs (fold change ≥ 2.0, P < 0.05).

A total of 4898 proteins with at least one unique peptide were identified with an FDR ≤ 1% (Fig 2A). Compared to the CON group, 378 proteins (300 upregulated, 78 downregulated) were identified to have significantly differential abundance in the TB group with fold change ≥ 1.5 (TB group/CON group protein abundance ratio ≥ 1.5 or ≤ 0.67) and P value less than 0.05 (*p* < 0.05) (Fig 2B), with 58 proteins increasing twofold or more and 51 proteins decreasing twofold or more. The heatmap showed the significant changes in proteins between the two groups. Selected proteins with fold change ≥ 2.0 in expression were annotated, and were shown in different colors on the heatmap (Fig 2C).

### Functional annotation of the differential expression proteins

**Fig 3:**
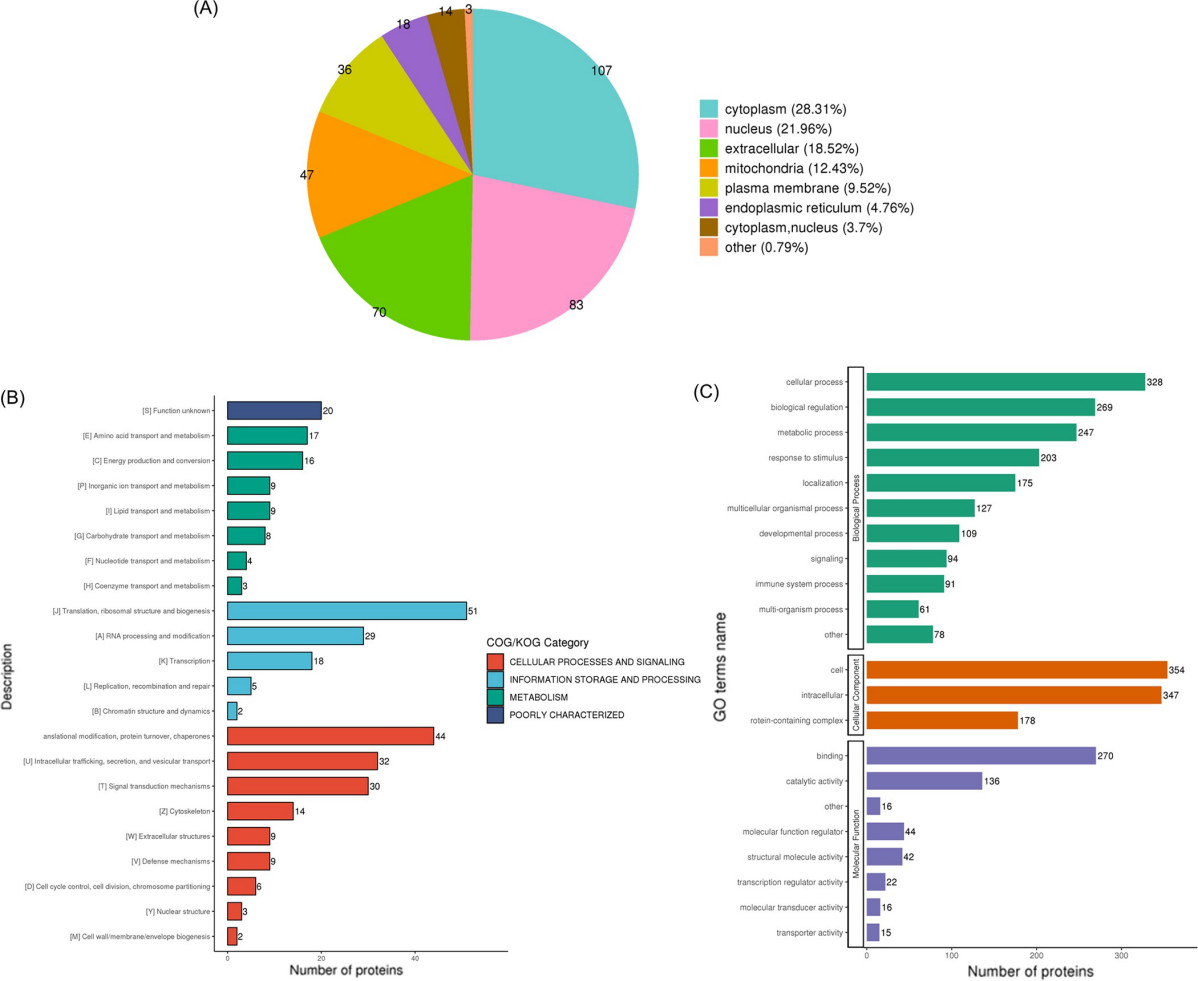
Functional annotation of the differentially expressed proteins in patients. (A) subcellular localizations; (B) COG / KOG functional classification statistics; (C) biological process, cellular component and molecular function.

Software Wolfpsort was used to predict protein subcellular localization, and data has indicated that, the differential expression proteins (DEPs) were localized in cytoplasm (28.31%), nucleus (21.96%), extracellular space (18.52%), mitochondria (12.43%), plasma membrane (9.52%), endoplasmic reticulum (4.76%), both nucleus and cytoplasm (3.7%), and others (0.79%) (Fig 3A).

COG / KOG functional classification statistics revealed that the main canonical pathways in which the DEPs participated included intracellular trafficking, secretion, and vesicular transport; posttranslational modification, protein turnover, chaperones; translation, ribosomal structure and biogenesis (Fig 3B).

In addition, GO function annotation was performed to evaluate the DEPs (Fig 3C). In molecular function, the main functional categories are binding, catalytic activity, molecular function regulator and structural molecule activity. In cell components, intracellular and protein—containing complex were the primary locations where DEPs were found. In biological processes, cellular process, biological regulation, metabolic process, response to stimulus and localization have changed significantly, and we noted that the "immune system process" category contains a total of 91 DEPs. Several DEPs associated with the inflammatory response, including MMP9, ELANE, CEACAM8, CTSG, were specifically down-regulated, indicating the inflammatory process was inhibited.

### GO and KEGG analysis of the differential expression proteins

**Fig 4:**
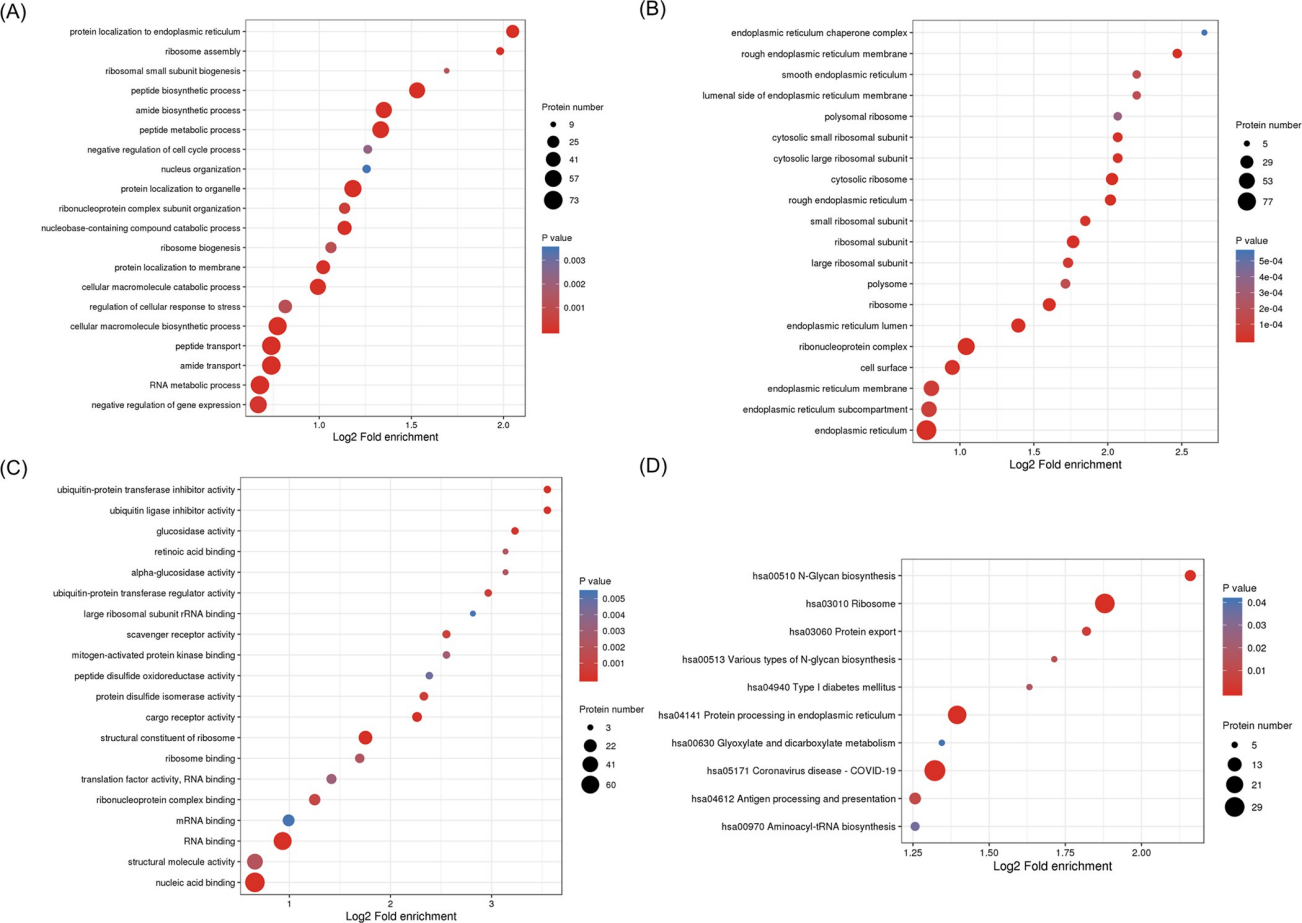
GO and KEGG analysis of the DEPs. (A) Biological process of DEPs. (B) Cellular component of DEPs. (C) Molecular function of DEPs. (D) KEGG pathway. The color of the dot stands for the different P-value and the size of the dot reflects the number of target genes enriched in the corresponding pathway.

For an overview of the function of all detected DEPs, a GO enrichment analysis was performed. The DEPs were classified into the biological process (Fig 4A), cellular component (Fig 4B), and molecular function (Fig 4C) categories. Most proteins were located in the rough endoplasmic reticulum membrane, cytosolic ribosome, ribosomal subunit, and endoplasmic reticulum lumen. Most DEPs were involved in nucleic acid binding, structural molecule activity and RNA binding. The DEPs were associated with regulation of cellular response to stress, protein localization to endoplasmic reticulum, peptide biosynthetic process, peptide transport, RNA metabolic process, and negative regulation of gene expression. The proteome associated with the cellular response to stress was significantly altered, which suggests that the fascia may have adaptively regulated in response to injury.

The KEGG pathway analysis was performed to better understand the functions of the DEPs. According to our findings, the DEPs primarily engage in 4 processes (Fig 4D), including Protein processing in endoplasmic reticulum (hsa04141), Ribosome (hsa03010), Aminoacyl−tRNA biosynthesis (hsa00970) and N−Glycan biosynthesis (hsa00510). Other enriched KEGG functional pathways were also found, such as Protein export, Type I diabetes mellitus, Coronavirus disease COVID−19, and Antigen processing and presentation. Genes involved in protein synthesis and export were found increased in the TB group, which indicated that there might be increased protein synthesis process in fascia tissue in fracture patients with blister.

### The interrelationship of the enriched pathways

**Fig 5:**
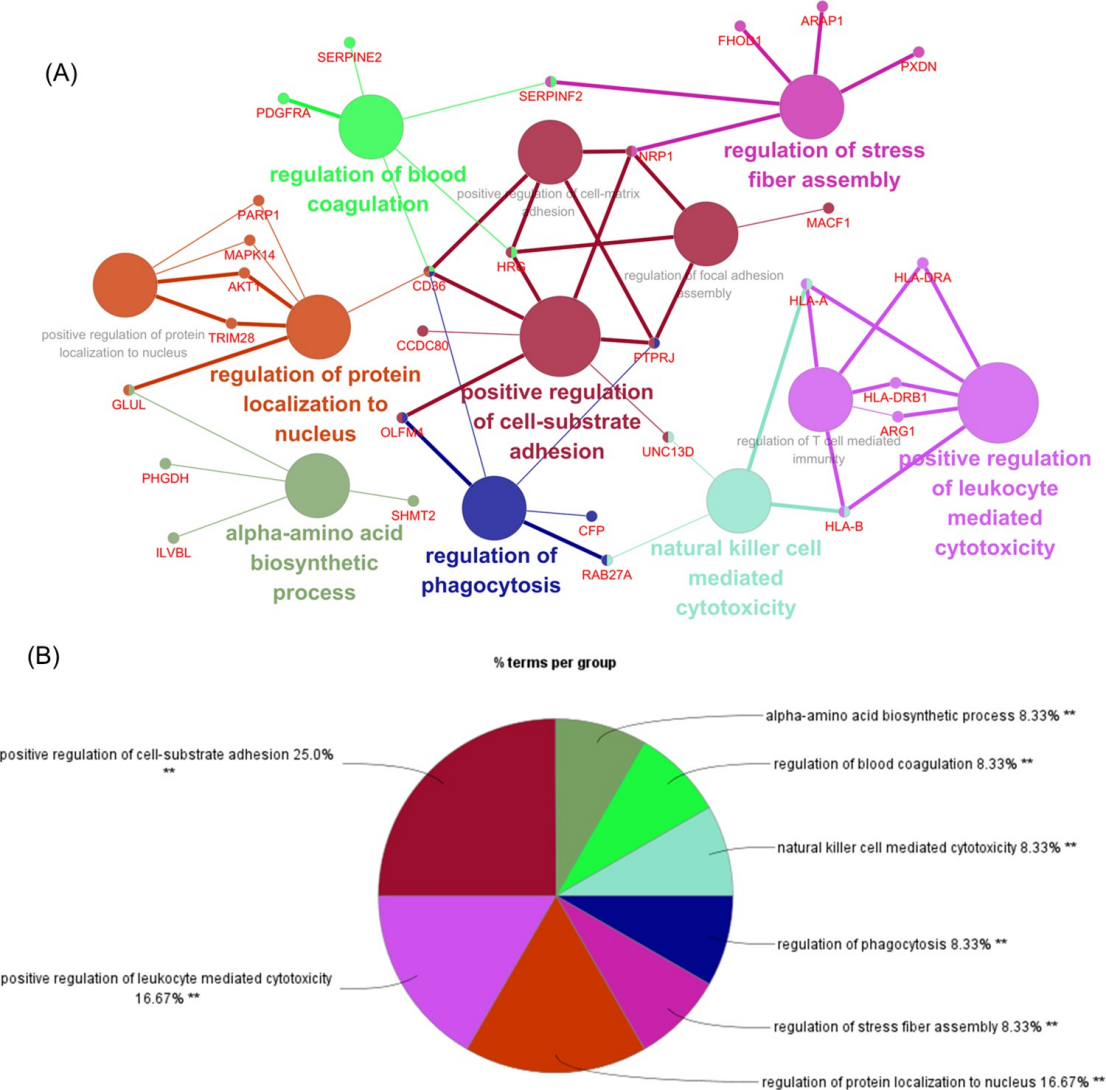
ClueGO enrichment analysis. (A) The interaction network of GO terms generated by the Cytoscape plug-in ClueGO. The significant term of each group is highlighted. (B) Proportion of each GO terms group in the total. GO, gene ontology. **P < 0.01.

The interrelationship of the enriched pathways was explored by GO (gene ontology) analysis. The screen was based on *p*<0.05 to visualize the results. From the ClueGO analysis, it can be visualized that the main enrichment is in the following pathways including the positive regulation of cell-substrate adhesion, regulation of stress fiber assembly, regulation of protein localization to nucleus, alpha-amino acid biosynthetic process, regulation of phagocytosis, positive regulation of leukocyte mediated cytotoxicity, natural killer cell mediated cytotoxicity, and regulation of blood coagulation (Fig 5A). The ratio of each GO term group was calculated (Fig 5B). Data from this analysis has indicated there might be a fiber re-architecture and immune response processes in fascia tissue of fracture patients with blister.

### Protein–protein interaction (PPI) network of the DEPs

**Fig 6:**
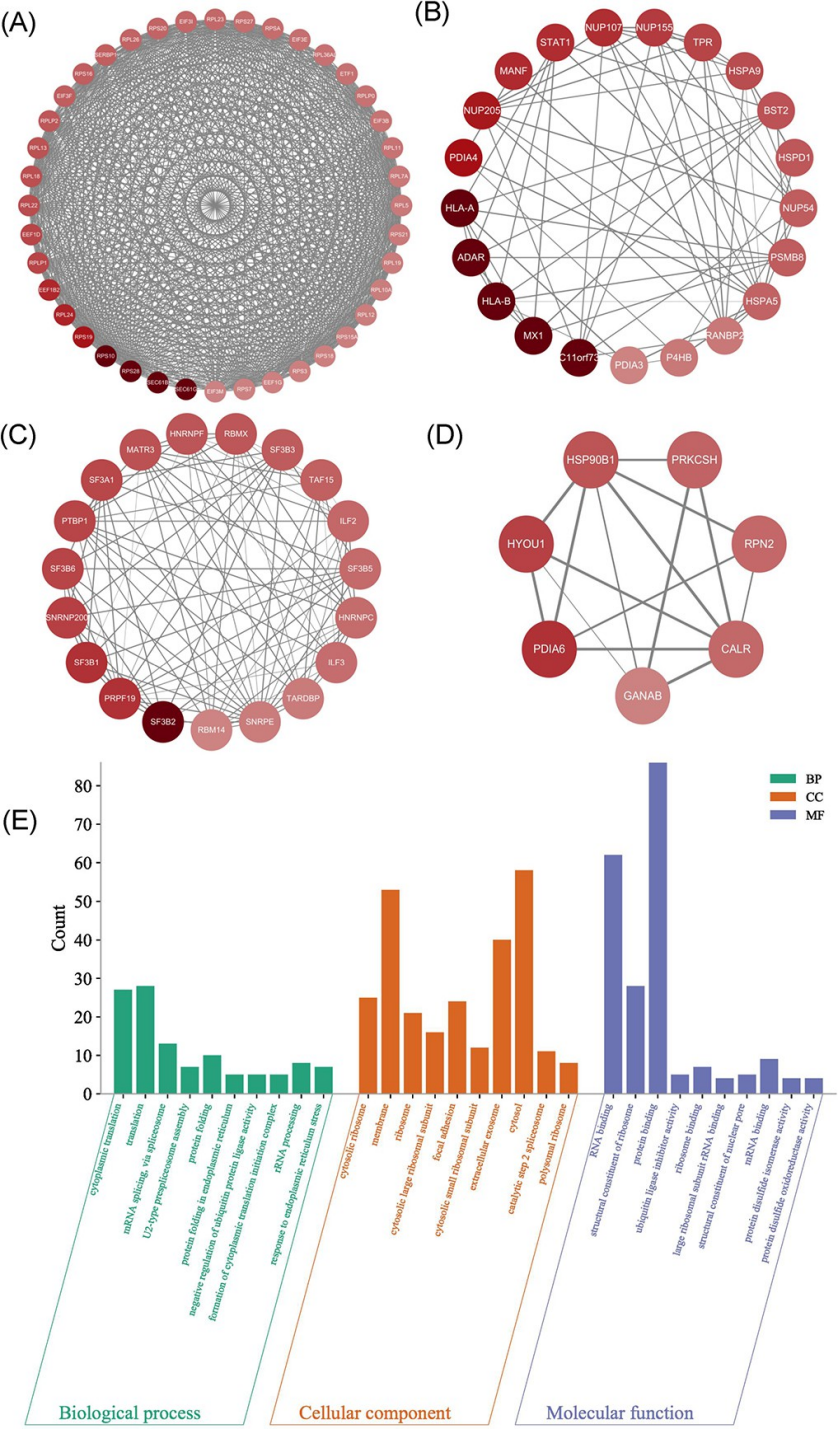
Protein-protein interaction network construction and the GO analysis. (A–D) Four clusters by the Cytoscape plug-in MCODE method of the DEPs. The nodes meant proteins. The edges meant the interaction of proteins. The darker the color, the greater the multiple of difference. The thicker the edge, the larger the combined score. (E) GO analysis of the DEPs selected by PPI.

A PPI network including DEPs was obtained from the STRING database [16], using a confidence score > 0.7 as the significance threshold. Cytoscape plug-in MCODE method was used to visualize the network (Fig 6A–6D). The data suggested that DEPs were mainly involved in pathways of formation of cytoplasmic translation initiation complex, mRNA splicing, rRNA processing, translation, protein folding and response to endoplasmic reticulum stress (Fig 6E), indicating elevated protein synthesis in fascia tissue in fracture patients with blister.

### Verification of differential proteins

**Fig 7:**
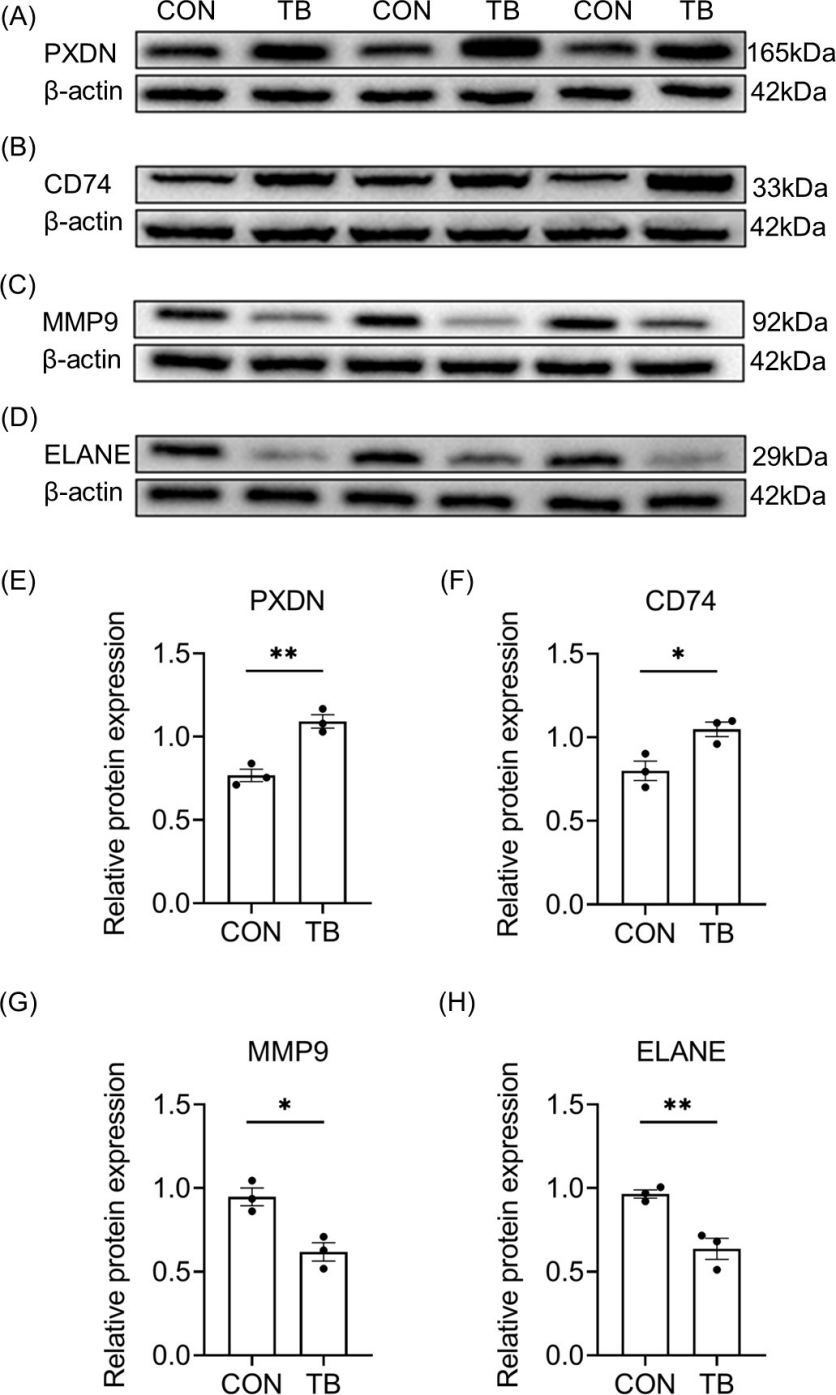
Confirmation of differential proteins. The protein expressions of PXDN (A and E), CD74(B and F), MMP9(C and G), and ELANE (D and H) of the deep fascia tissues were measured by Western blot. *P <0.05, **P<0.01 vs. CON. N = 3.

Compared to those in the CON group, expressions of PXDN and CD74 were significantly increased while the expression of MMP9 and ELANE were significantly decreased in fascia tissue from TB patients (*p*<0.05, Fig 7A–7H), which was consistent with the results from proteomics analysis. These data indicated that the fascia tissue might undergo microenvironment reconstruction that was adaptive to the increasing compartment pressure during ACS.

## Discussion

The most significant contribution of this study is to provide more evidence for diagnosis and treatment strategies of ACS by exploring the role of fascia in the pressure release process. ACS is defined as a critical pressure increase within a confined compartmental space causing a decline in the perfusion pressure to the tissue within that compartment [17]. It most commonly occurs following limb fracture or crush injury. It has been traditionally considered that, due to the limited elasticity of the deep fascia, increased pressure in fascial compartment caused by the tissue injury may consequently result in ischaemic necrosis of the muscles and nerves [18]. However, it is noteworthy that, in patients with lower leg fractures, following the appearance of tension blisters on the injured limb, some ACS symptoms such as pain and paresthsia are alleviated and the fascial compartment pressure is reduced simultaneously. In a retrospective analysis, Guo et al found that among patients who developed severe tibial plateau fractures, those who developed tension blisters in the affected limb had a lower risk of ACS [19], suggesting that there might be some pressure-releasing mechanisms which can protect tissues from the detrimental consequences of ACS. This hypothesis is also termed as "myofascial self-release law" [5].

Deep fascia, as an important structure in the development of ACS, most likely plays an important role in the process of pressure release within the fascial compartment. Evidence has indicated that, fascia may be more than a connective tissue with a protective wrapping effect, it could reduce friction between muscles, transmit mechanical forces generated by the musculoskeletal system, and harmonize movement [20–22]. Previous studies have found that there are mechanical interactions between muscle, fascia and skin [23–25], and that when muscle contracts, the force can be transmitted outwards through the fascial system, causing skin stretch or wrinkle [26, 27]. Wilke et al. demonstrated that the transmission of myofascial forces could be affected by the thickness and the collagen content of fascia; and thicker fascia with higher collagen content may enhance the tensile strength of myofascia, thereby facilitating myofascial force transmission [28]. As the primary cells in the deep fascia, fibroblasts may regulate the structure and the tension of the deep fascia by remodeling fibrin and matrix components [29, 30]. Elastic fibers play an important role in the elastic capacity of deep fascia [31]. Therefore the fibrous components achieve force transmitting, dynamic position modulating and energy transmission [32]. The organization of stress fibers is generally considered to be a structural adaptation that allows mechanically stretched cells to adjust their shapes and orientations, which plays a crucial role in maintaining cellular mechano-homeostasis under stretching conditions [33–36]. In this preliminary study, we were the first to perform LC-MS/MS label-free quantitative proteomics to compare the protein profile of deep fascia tissue in patients with lower leg fractures with and without tension blisters. The pathway involved in the regulation of stress fiber assembly was enriched by GO analysis in this study, suggesting that there may be adaptive regulation of the deep fascia in response to the increased fascial tension and cellular stretch caused by progressively elevated pressure in the compartment during the progression of ACS.

In our previous study, we found that collagen fibers in the deep fascia were broken in the early stage of ACS, which may be a result of the deep fascia self-regulation and the release of intracompartmental pressure [37]. In the present study, we found further that the expression of PXDN (promoting the function of fibroblasts [38]) and CD74 (enhancing the proliferation process of fibroblasts [39]) were increased in the deep fascia tissue of patients with blister (*p* all <0.05). According to the GO enrichment analysis and the KEGG-based functional enrichment analysis, the majority of DEPs were found to be located in the rough endoplasmic reticulum membrane, and the DEPs are involved in pathways related to protein synthesis and processing. In addition, expression of MMP9 (involved in collagen hydrolysis [40]), and ELANE (involved in elastin hydrolysis [41]), were all significantly reduced (*p* all <0.05), suggesting that fibroblasts might participate in the repair of damaged fascial tissue by promoting cell proliferation and collagen synthesis. Reduced hydrolysis of collagen and elastin may modulate the stiffness and elasticity of deep fascia and promote its recovery by repairing broken collagen fiber bundles. Therefore, the appearance of tension blisters on the skin might be the sign of local pressure release from the fascial compartment. It might be possible that deep fascia could achieve self-modulation under ACS by re-constructing its structure, releasing the pressure, avoiding further damage, and self-repair.

The present study still has some limitations. First, all samples from patients with blister were collected during the operation, which could not provide the dynamic changes in gene expression profile following the ACS development. Secondly, the samples size was relatively small. Thirdly, no pressure data was provided in this study due to lack of standardized devices for non-invasive dynamic intracompartmental pressure measurement.

## Conclusions

In the fascia tissue of ACS patients with blisters, the expression of PXDN and CD74 were upregulated, while the expression of MMP9 and ELANE were reduced, indicating fascia tissue underwent microenvironment reconstruction during ACS. Understanding the underlying mechanisms of deep fascia in ACS patients will provide more evidence for the accurate diagnosis, proper therapeutic strategies and the disease prognosis.

## Supporting information

S1 Raw images (PDF)

S1 Graphical abstract (TIF)

## Abbreviations

ACS: Acute compartment syndrome
LC-MS/MS: Liquid chromatography-tandem mass spectrometry
FDR: False discovery rate
GO: Gene ontology
COG/KOG: Clusters of orthologous groups of proteins/Eukaryotic ortholog groups
KEGG: Kyoto encyclopedia of genes and genomes
PXDN: Peroxidasin homolog
CD74: Leukocyte differentiation antigen 74
MMP9: Matrix metalloproteinase-9
ELANE: Neutrophil elastase
DEPs: Differential expression proteins
