# Supplementary figures and images for: Proteomics analysis of deep fascia in acute compartment syndrome
Source: PLoS One. 2024 Jul 1;19(7):e0305275. doi: 10.1371/journal.pone.0305275 (PMC11216580; doi:10.1371/journal.pone.0305275)

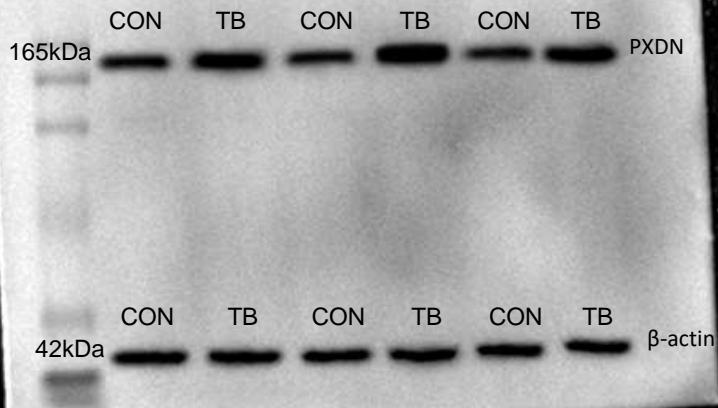

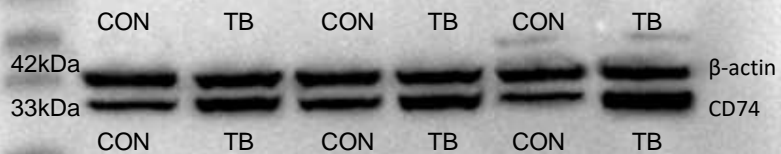

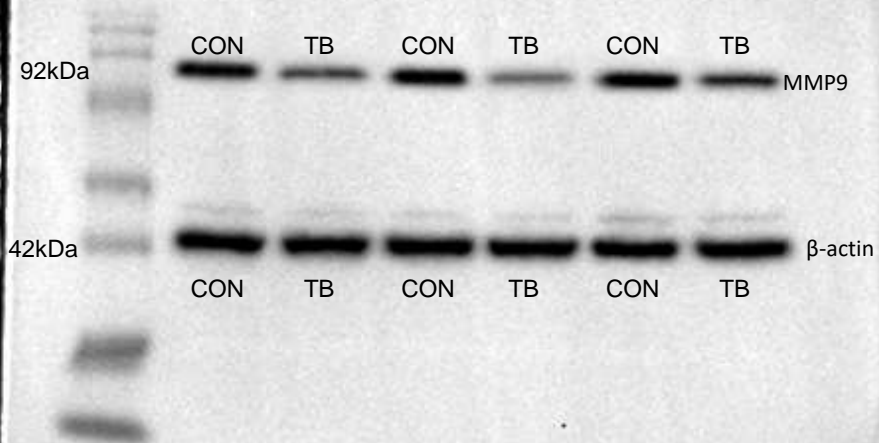

42kDa

CON

TB

CON

TB

CON

TB

$\beta$ -actin

29kDa

CON

TB

CON

TB

CON

TB

ELANE

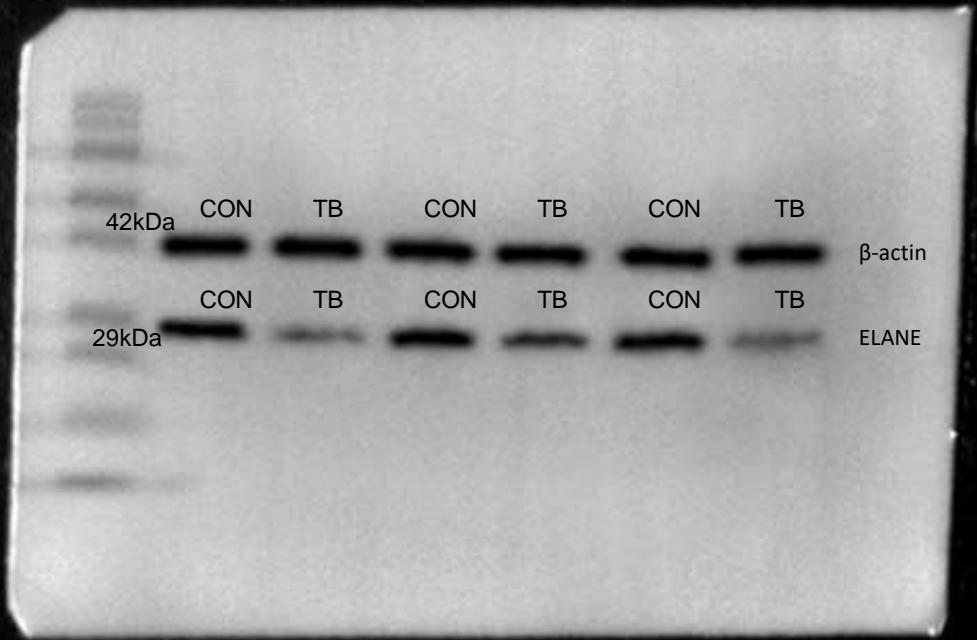

Supplement: S1 Raw images — (PDF) [file pone.0305275.s001.pdf]

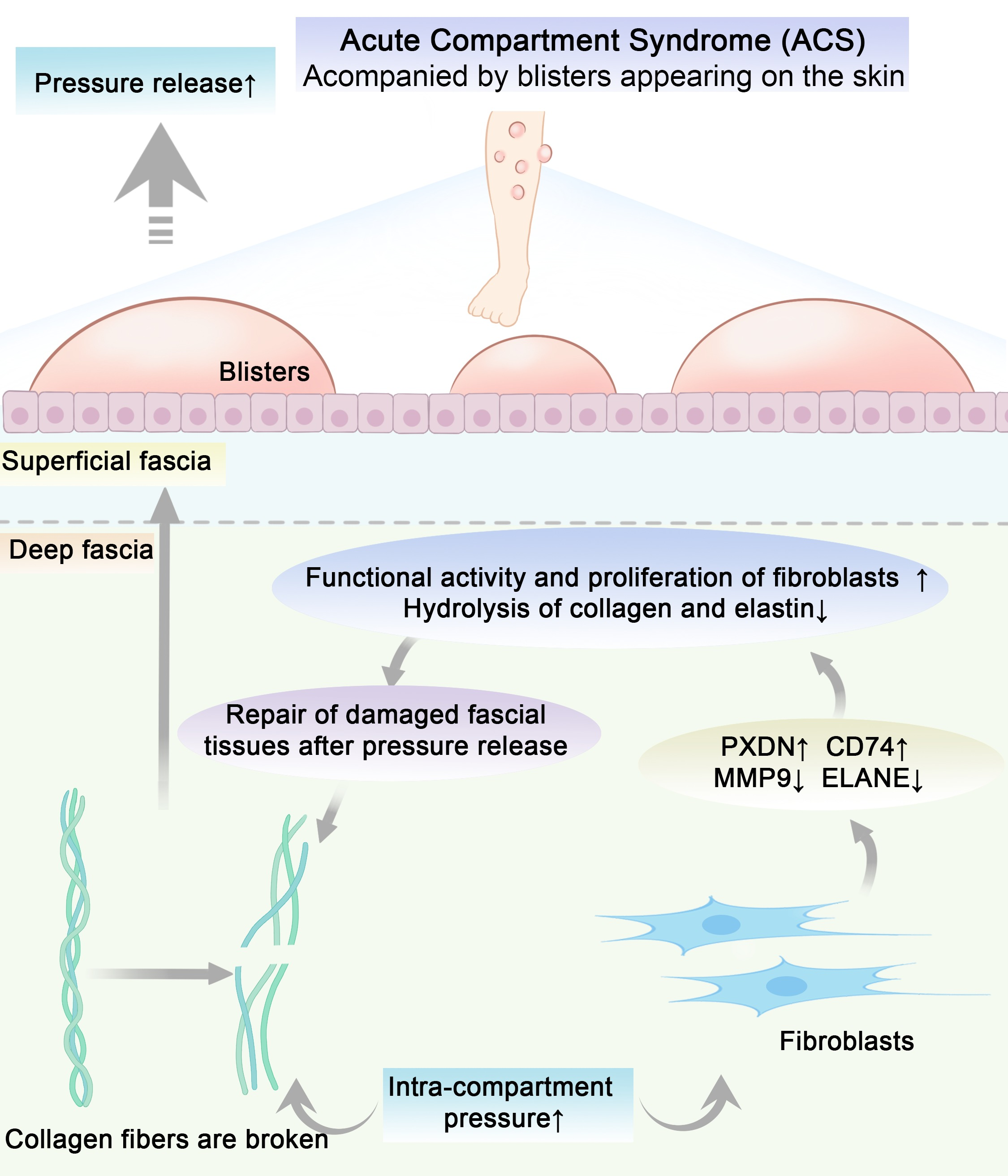

Supplement: S1 Graphical abstract — (TIF) [file pone.0305275.s002.tif]
